# The normative values of pain thresholds in healthy Taiwanese

**DOI:** 10.1002/brb3.3485

**Published:** 2024-04-22

**Authors:** Li‐Ling Hope Pan, Yu‐Hsiang Ling, Kuan‐Lin Lai, Yen‐Feng Wang, Fu‐Jung Hsiao, Shih‐Pin Chen, Hung‐Yu Liu, Wei‐Ta Chen, Shuu‐Jiun Wang

**Affiliations:** ^1^ Brain Research Center National Yang Ming Chiao Tung University Taipei Taiwan; ^2^ College of Medicine National Yang Ming Chiao Tung University Taipei Taiwan; ^3^ Department of Neurology Neurological Institute Taipei Veterans General Hospital Taipei Taiwan; ^4^ Institute of Clinical Medicine National Yang Ming Chiao Tung University Taipei Taiwan; ^5^ Department of Medical Research Taipei Veterans General Hospital Taipei Taiwan; ^6^ Department of Neurology Keelung Hospital, Ministry of Health and Welfare Keelung Taiwan

**Keywords:** cold pain threshold, Eastern Asian, healthy adult, heat pain threshold, mechanical punctate pain threshold, pressure pain threshold

## Abstract

**Objective:**

Quantitative sensory testing is widely used in clinical and research settings to assess the sensory functions of healthy subjects and patients. It is of importance to establish normative values in a healthy population to provide reference for studies involving patients. Given the absence of normative values for pain thresholds in Taiwan, the aim of this study was to report the normative values for future reference in the Taiwanese population and compare the differences between male and female participants.

**Methods:**

Healthy adults without any chronic or acute pain condition were recruited. The pain thresholds were assessed over the cephalic (supraorbital area and masseter muscle) and extracephalic (medio‐volar forearm and thenar eminence) areas. The heat, cold, mechanical punctate, and pressure pain thresholds were measured with a standardized protocol. Comparisons between male and female participants were performed.

**Results:**

One hundred and thirty healthy participants (55 males: 30.4 ± 7.4 years; 75 females: 30.5 ± 8.1 years) finished the assessments. Male participants were less sensitive to mechanical stimuli, including pressure over masseter muscle (male vs. female: 178.5 ± 56.7 vs. 156.6 ± 58.4 kPa, *p* = .034) and punctate over medio‐volar forearm (male vs. female: 116.4 ± 45.2 vs. 98.7 ± 65.4 g, *p* = .011), compared to female participants. However, female participants were less sensitive to cold stimuli, indicated by lower cold pain thresholds over the supraorbital area (male vs. female: 18.6 ± 8.4 vs. 13.6 ± 9.3°C, *p* = .004), compared to male participants. No significant differences were found between sexes in other pain threshold parameters.

**Conclusions:**

We provided the normative values of healthy male and female adults in Taiwan. This information is crucial for comparison in future pain‐related studies to identify potential hypoalgesia or hyperalgesia of tested subjects.

## Graphical abstract

This is the first study that reports the normative values of pain thresholds following a standardized protocol of quantitative sensory testings among a large healthy cohort without any pain conditions in East Asia. These results are crucial as a comparison for future pain‐related research.

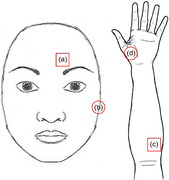

## 1 INTRODUCTION

Quantitative sensory testing (QST) is one of the most commonly used tools to assess sensory functions. It has been widely used both clinically and in research. German Research Network on Neuropathic Pain (DFNS) developed and published the standardized clinical protocol (Rolke, Magerl, et al., 2006) for QST including instructions for the investigators. The QST protocol was specifically designed to assess the function of the primary afferent fibers, including Aβ, Aδ, and C fibers (Backonja et al., 2013). Beside assessing sensory functions, QST can serve as outcome predictors (Georgopoulos et al., 2019; Pan, Wang, Ling, et al., 2022; Schliessbach et al., 2018), observational parameters (Greenspan et al., 2020; Krimmel et al., 2022; Pan, Wang, Lai, et al., 2020), and so forth in disorders associated with sensory functions. Migraine is one of the most common neurological disorders that can be considered as sensory threshold disease (Peng & May, 2019). The prevalence of migraine is approximately 15% worldwide and yield a high burden as migraine accounts for 4.9% of global ill health (Steiner & Stovner, 2023). One manifestation of migraine is cutaneous allodynia, defined as “pain due to a stimulus that does not normally provoke pain,” which can serve as a predictor of migraine chronification (Louter et al., 2013). Parameters of QST quantitatively assess the cutaneous allodynia in migraine. Migraine is a dynamic disease with “on” and “off” phases. Previous studies using QST have found changes in pain sensitivities during different phases of migraine and considered increased pain sensitivities as a predictor of migraine attack (Pan, Wang, Lai, et al., 2020; Sand et al., 2008; Scholten‐Peeters et al., 2020; Schwedt et al., 2015). Hence, the investigation of pain sensitivity using QST posits clinical significance in studies with migraine and the establishment of normative values is of importance.

The reference data of QST were reported by the DFNS (Magerl et al., 2010; Rolke, Magerl, et al., 2006). Some other research groups also provided reference data for healthy subjects of Caucasians (Knutti et al., 2014; Nothnagel et al., 2017), Hispanic Latinos (González‐Duarte et al., 2016), or African Americans (Powell‐Roach et al., 2019) from various body sites. However, previous studies have also demonstrated that pain perception or pain thresholds differ between ethnicities (Aufiero et al., 2017; Herbert et al., 2017; Ostrom et al., 2017; Yang et al., 2013). To the best of our knowledge, the normative values for pain thresholds in the Eastern Asian population with a large cohort are still lacking. There was only one study (Wang et al., 2014) done in the Chinese population with 20 subjects reporting the reference data. Therefore, the aim of the study was to provide normative values for the healthy Eastern Asian population, more specifically, the healthy Taiwanese population. Furthermore, we compared the differences in pain thresholds between male and female participants.

## 2 METHODS

### 2.1 Participants

We recruited healthy participants aged between 20 and 50 years old without (a) history of major systemic illness, such as uncontrolled hypertension, diabetes, chronic kidney disease, autoimmune disease, or malignancy; (b) history of neurological disorders that may alter sensation, such as stroke or peripheral neuropathy; (c) history of psychiatric illness, for example, anxiety disorders and major depression or regular use of psychotropic agents, such as antidepressants, antipsychotics, or sedatives; (d) daily consumption of >20 cigarettes; (e) pregnancy or lactation; (f) any obvious infection or inflammation over a period of at least 1 month before enrollment; and (g) any localized inflammatory process or other active pathologies involving the forehead or the arm where QST was to be administered. This study was part of a broader, integrated project aimed at decoding of pain sensitivity through multiple modalities in healthy participants. The study protocol was approved by the Institutional Review Boards in Taiwan (IRB‐YM108044F and 2020‐11‐004C). All participants provided written informed consent prior to participation. The study procedures followed the Declaration of Helsinki. Data from this study are available from the corresponding author upon reasonable request.

### 2.2 QST

**FIGURE 1:**
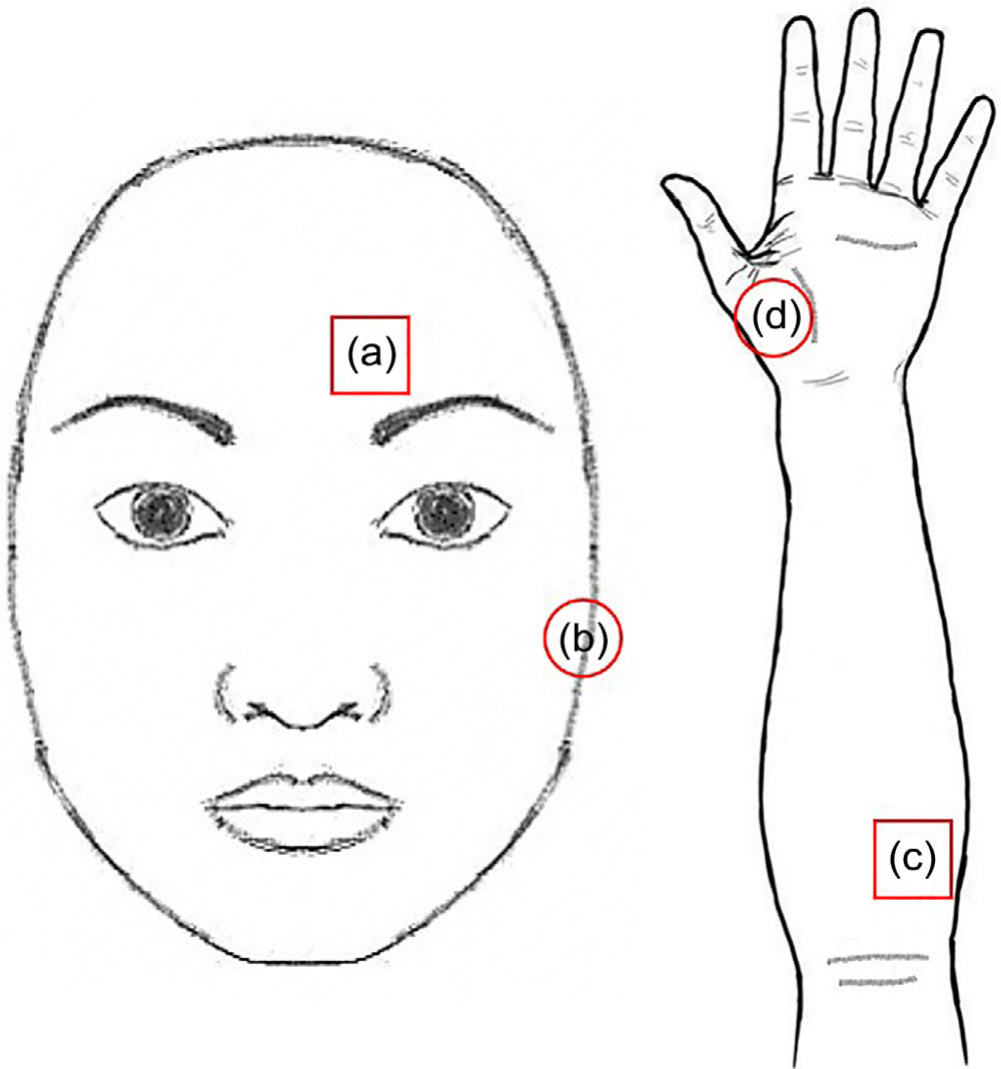
Testing sites. The testing sites for (a) cold, heat, and mechanical punctate pain threshold (MPT) over the forehead; (b) pressure pain threshold (PPT) over masseter muscle; (c) cold, heat, and MPT over the forearm; and (d) PPT over thenar eminence.

Participants were familiarized with the QST procedure by demonstrating the stimuli on right thenar eminence, which was not the testing target for QST assessment later. The participants lay comfortably in the supine position during the assessment. The left supraorbital (i.e., the first branch of the trigeminal nerve dermatome, V1, Figure 1a) and medio‐ventral forearm (i.e., the first thoracic nerve dermatome, T1, Figure 1c) areas were targeted except for pressure stimuli, which were applied on the muscle bellies of the masseter muscle (Figure 1b) and thenar eminence (Figure 1d). Most stimulus parameters were adopted from the DFNS protocol (Rolke, Baron, et al., 2006), except that all stimuli were given as ramps (method of limits) in order to make them more comparable to each other. The heat pain threshold (HPT), cold pain threshold (CPT), mechanical punctate pain threshold (MPT), and pressure pain threshold (PPT) were assessed in both dermatomes by experienced assessors among all participants. Studies with the same protocol of these assessments have been published (Pan, Wang, Lai, et al., 2020; Pan, Wang, Ling, et al., 2022). The room for QST assessment was air‐conditioned and well‐controlled at 22 to 23°C and humidity of 60%–70%.

#### 2.2.1 HPT and CPT

**FIGURE 2:**
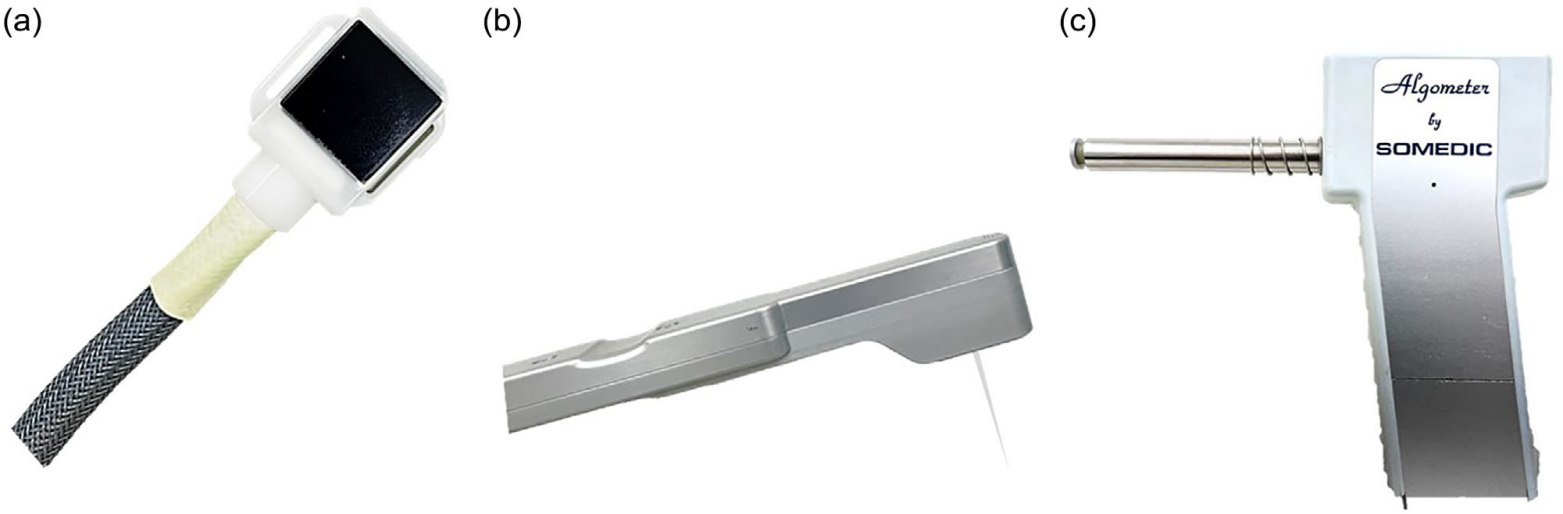
Modalities for thermal and mechanical stimuli. (a) Thermode for cold and heat stimuli, (b) electronic von Frey filament for mechanical punctate stimuli, and (c) electronic algometer for pressure stimuli.

The HPT and CPT were determined using the Medoc TSA‐II NeuroSensory Analyzer (Medoc Ltd.) with a 30 × 30 mm Thermode (Figure 2a) placed on the testing area skin and secured with Velcro straps. To determine the HPT, five heat stimuli, starting at 32 ± 0.5°C with a cut‐off temperature of 50°C, were given at the increasing rate of 1°C per second; and to determine CPT, five cold stimuli, starting at 32 ± 0.5°C with a cut‐off temperature at 0°C, were given at the decreasing rate of 1°C per second (Rolke, Baron, et al., 2006). The HPT or CPT was defined as the lowest/highest temperature that the participants perceived as painful.

#### 2.2.2 MPT

Mechanical punctate stimuli were given with the standard rigid electronic von Frey filament (BIO‐EVF5, BioSeb; Figure 2b). The MPT was defined as the lowest intensity perceived as painful for participants. The assessors applied steady force, approximately at the rate of 25 g/s (Reitz et al., 2016), with the electronic von Frey filament over the testing sites. Participants were instructed to inform the assessor immediately upon painful sensation.

#### 2.2.3 PPT

The pressure stimuli were applied over muscles as suggested by the DFNS protocol (Rolke, Magerl, et al., 2006) on the muscle bellies of the masseter muscle (Figure 1b) and thenar eminence (Figure 1d). The pressure stimuli were given by a 1‐cm^2^ probe attached to a hand‐held electronic algometer (Algometer Type II, SBMEDIC Electronics; Figure 2c). The PPT was defined as the lowest intensity perceived as painful for participants. The assessors applied steady force, approximately at the rate of 50 kPa/s (Rolke, Baron, et al., 2006), with an electronic algometer over the testing sites. Participants were instructed to inform the assessor immediately upon painful sensation.

### 2.3 Statistical analyses

Kolmogorov–Smirnov tests were used to assess whether the data were normally distributed. Differences between sexes were compared using independent *t*‐tests or Mann–Whitney *U* tests for all QST parameters. The significant level was set at *p* < .05. All statistical analyses were performed by the Statistical Package for Social Sciences version 24 (SPSS, IBM).

## 3 RESULTS

One hundred and thirty healthy participants (55 males) completed the QST assessment. Table 1 shows the demographic data of male and female participants. No significant age difference was found between male and female (30.4 ± 7.4 vs. 30.5 ± 8.1 years, *p* = .901) Male participants showed significantly higher body mass index compared to females (24.3 ± 3.9 vs. 22.4 ± 2.8 kg/m^2^, *p* = .005); however, they were around the average (male: 25.4 kg/m^2^, female: 23.1 kg/m^2^) of adult (19–44 years group) Taiwanese population reported by the Ministry of Health and Welfare (Ministry of Health & Welfare, 2016).

**TABLE 1:** Demographic data and pain thresholds of male and female participants.

		Male	Female	*p*
*N*		55	75	–
Age (years)		30.4 ± 7.4	30.5 ± 8.1	.901^c^
BMI (kg/m^2^)		24.3 ± 3.9	22.4 ± 2.8	.005^#^
V1	CPT (°C)	17.3 ± 9.0	16.1 ± 8.8	.471^c^
	HPT (°C)	42.6 ± 3.4	42.6 ± 3.8	.940
	MPT (g)	121.1 ± 47.3	112.7 ± 45.7	.368^c^
	PPT^a^ (kPa)	178.5 ± 56.7	156.6 ± 58.4	.034^*^
T1	CPT (°C)	18.6 ± 8.4	13.6 ± 9.3	.004^#^
	HPT (°C)	40.8 ± 3.7	41.1 ± 3.8	.838^c^
	MPT (g)	116.4 ± 45.2	98.7 ± 65.4	.011^#^
	PPT^b^ (kPa)	290.3 ± 91.3	256.7 ± 94.8	.064^c^

Abbreviations: BMI, body mass index; CPT, cold pain threshold; HPT, heat pain threshold; MPT, mechanical punctate pain threshold; PPT, pressure pain threshold; T1, first thoracic nerve; V1, first branch of trigeminal nerve.

^a^ Testing site on masseter muscle.

^b^ Testing site on thenar eminence.

^c^ Calculated with Mann–Whitney *U* tests.

^*^ *p* < .05 with independent t tests.

^#^ *p* < .05 with Mann–Whitney *U* tests.

### 3.1 QST results

Table 1 shows the four different parameters of males and females over two different testing areas. Significant differences between male and female were found in masseter PPT (male vs. female: 178.5 ± 56.7 vs. 156.6 ± 58.4 kPa, *p* = .034), T1 CPT (male vs. female: 18.6 ± 8.4 vs. 13.6 ± 9.3°C, *p* = .004), and T1 MPT (male vs. female: 116.4 ± 45.2 vs. 98.7 ± 65.4 g, *p* = 0.011) where male participants were more sensitive to cold pain and less sensitive to mechanical pain as compared to female participants; trend of higher thenar PPT (male vs. female: 290.3 ± 91.3 vs. 256.7 ± 94.8 kPa, *p* = .064) was also found in male as compared to female. Table S1 shows the raw data and 95% confidence intervals for each parameter of male and female participants.

## 4 DISCUSSION

This current study provides normative pain threshold values in healthy Taiwanese populations free from any acute or chronic pain conditions. These normative values are crucial for future studies involving pain as a comparison since the currently available data were mostly from Caucasians. Understanding the normative value from the healthy cohort of the same population could provide clinicians with objective reference values when examining sensory functions in clinical settings.

The results of our study showed that males were more sensitive to cold‐induced pain, compared to females, over the forearm. On the other hand, males were less sensitive to mechanical pain, compared to females, in both punctate and pressure pain. Our findings are different from the studies conducted in the European/Caucasian population that showed males being less sensitive than females regarding thermal pain (Magerl et al., 2010; Rolke, Baron, et al., 2006). However, the testing sites were slightly different in these studies; the previous studies reported results from the cheek and dorsal hand, whereas our study tested on the supraorbital area and medio‐ventral forearm. Yet, it is still interesting to dig into the reason why males in our current population were more sensitive to cold. The exact underlying reason is unknown; however, we could provide some possible explanations. These previous studies were conducted in several cities in Germany, including Mainz, Munich, Ulm, and so forth. These cities are all located at higher latitudes with the lowest mean monthly temperature around 0°C or below in January. Residents in these cities are familiar with snow and cold. On the other hand, Taipei, where our study was conducted, is a city located at a lower latitude with the lowest mean monthly temperature around 13°C in January. Cold spell is rare let alone snow. Residents in Taipei may be less familiar or sentient to the change of cold. Furthermore, transient receptor potential cation channel subfamily M member 8 (TRPM8) is widely recognized as having a central role in cold sensation (Bautista et al., 2007) and *TRPM8* is expressed in cold pain and temperature‐sensitive neurons of the dorsal root ganglia (Peier et al., 2002). A recent study discovered divergent roles of *TRPM8* in pain modulation between male and female mice (Alarcón‐Alarcón et al., 2022). In addition, a study has also found one of the *TRPM8* variants, rs10166942, demonstrated a gradient relationship with latitude (Key et al., 2018), with frequencies ranging from 5% in Nigeria (average annual temperature: 28°C) to 88% in Finland (average annual temperature: 6°C). The potential influence of ethnicity or latitude of residence on the modulatory role of *TRPM8* in different sexes remains unknown. Unfortunately, based on the current study design, we cannot rule out the influence of the cultural or environmental background. Whether the climate or the familiarity with cold influences the interpretation of cold‐induced pain remains unknown and would be an exciting topic to explore. Future studies investigating deeper into life experiences or personal traits may provide more insights into this matter.

Table 2 shows the comparison of our results with others (Dyck et al., 1993; González‐Duarte et al., 2016; Powell‐Roach et al., 2019; Rolke, Baron, et al., 2006; Waller et al., 2016; Wang et al., 2014; Wasner & Brock, 2008). MPT was not listed because most studies used traditional von Frey filaments, which are not comparable to the results from the electronic von Frey filament employed in our study. The testing locations were grouped by their optimal proximity. Our cohort showed higher pressure pain sensitivities compared to the two studies reporting PPTs (Rolke, Baron, et al., 2006; Waller et al., 2016). In terms of thermal pain thresholds, CPT exhibits more significant variance across studies, with the largest difference of 13.6°C, whereas HPT shows less variance, with the largest difference being 7°C. Our CPTs and HPTs demonstrated thermal sensitivities akin to the study involving Hispanic participants (González‐Duarte et al., 2016) and higher sensitivities, compared to studies predominantly involving Caucasians (Rolke, Baron, et al., 2006; Waller et al., 2016; Wasner & Brock, 2008), and lower sensitivities, compared to African Americans (Powell‐Roach et al., 2019). This aligns with findings from a previous review (Kim et al., 2017) that suggest that ethnicity may affect pain thresholds, indicating ethnic minorities commonly had lower pain thresholds, even without statistical significance. Moreover, the latitude of the residence may also interfere with the pain sensitivity, as evidenced by the aforementioned cold‐pain‐related gene, *TRPM8*, which demonstrated a gradient relationship with latitude (Key et al., 2018). Further investigation is warranted to comprehend the interplay between ethnicity, residence, and pain sensitivities.

**TABLE 2:** Comparison of values between our study and other studies.

		Pan	Rolke	Gonzalez‐Duarte	Powell‐Roach	Dyck	Wasner	Wang	Waller
Country/region		Taipei, Taiwan	10 centers in Germany	Mexico City, Mexico	Chicago, USA^a^	Rochester, USA	Sydney, Australia	Nanjing, China	Western Australia
Face	CPT (°C)	17.6/16.1	14.7/18.4	–	–	–	–	16.2	–
	HPT (°C)	42.6/42.6	43.7/41.5	–	–	39.0	–	41.9	–
	PPT (kPa)	179/157	226/202	–	–	–	–	–	–
Forearm/hand	CPT (°C)	18.6/13.6	12.5/16.2	19.7	25.4/27.2	–	17.7/20.3	16.2	13.7
	HPT (°C)	40.8/41.1	44.1/42.6	42.7	38.5/37.1	40.4	43.9/43.5	43.0	–
	PPT (kPa)	290/258	424/350	–	–	–	–	–	360

*Note*: Data resented as the mean of male/female or mean of pooled subjects.

^a^ Study targeted on Black/African American.

The study has limitations. We did not recruit participants aged over 50. One important strength of our study was that we assessed pain sensitivities in those without “any acute or chronic pain condition.” Considering that pain conditions are more common in the senior population due to arthritis or other degenerative conditions, we did not recruit participants over the age of 50. Yet, investigation of the pain thresholds in the senior population regardless of pain conditions can also be considered in the future. We did not repeat the assessment of the pain thresholds to investigate the test–retest reliability. Future investigation of this matter should be considered. We assess the pain thresholds only on the left. However, based on previous studies (O'Neill & O'Neill, 2015; Rolke, Baron, et al., 2006), no significant differences were found between the sides. Nonetheless, this is the first study that reports normative pain threshold values with a large sample size in Eastern Asia. We reported the pain sensitivity of healthy controls without any acute or chronic pain conditions, which can be made as reference data for future QST studies in similar population.

## AUTHOR CONTRIBUTIONS

**Li‐Ling Hope Pan**: Conceptualization; data curation; formal analysis; methodology; project administration; writing—original draft. **Yu‐Hsiang Ling**: Data curation; formal analysis; investigation; validation; writing—review and editing. **Kuan‐Lin Lai**: Conceptualization; project administration; writing—review and editing. **Yen‐Feng Wang**: Conceptualization; project administration; writing—review and editing. **Fu‐Jung Hsiao**: Conceptualization; project administration; writing—review and editing. **Shih‐Pin Chen**: Conceptualization; project administration; writing—review and editing. **Hung‐Yu Liu**: Conceptualization; project administration; writing—review and editing. **Wei‐Ta Chen**: Conceptualization; project administration; writing—review and editing. **Shuu‐Jiun Wang**: Conceptualization; funding acquisition; project administration; supervision; writing—review and editing.

## CONFLICT OF INTEREST STATEMENT

The authors declare no conflicts of interest.

### PEER REVIEW

The peer review history for this article is available at https://publons.com/publon/10.1002/brb3.3485.

## Supporting information

TABLE S1 Raw data and 95% confidence intervals for each parameter of male and female participants.

## Data Availability

Data from this study are available from the corresponding author upon reasonable request.
