# Supplementary material for: The normative values of pain thresholds in healthy Taiwanese
Source: Brain Behav. 2024 Apr 22;14(4):e3485. doi: 10.1002/brb3.3485 (PMC11034865; doi:10.1002/brb3.3485)
Supplement: Supplementary file 1 — TABLE S1 Raw data and 95% confidence intervals for each parameter of male and female participants. [file BRB3-14-e3485-s001.docx]

Table S1. Raw data and 95% confidence intervals for each parameter of male and female participants

|  | |  | V1 | | | | |  | T1 | | | | |
| --- | --- | --- | --- | --- | --- | --- | --- | --- | --- | --- | --- | --- | --- |
|  |  | | Mean | SD | 95% CI | |  | Mean | | SD | 95% CI | |  |
|  |  |  |  |  | lower | upper |  |  |  |  | lower | upper |  |
| male | CPT (℃) | | 17.3 | 9.0 | -0.3 | NA^c^ |  | 18.6 | | 8.4 | 2.1 | NA^c^ |  |
|  | HPT (℃) | | 42.6 | 3.4 | 36.0 | 49.2 |  | 40.8 | | 3.7 | 33.5 | 48.1 |  |
|  | MPT (g) | | 121.1 | 47.3 | 28.5 | 213.8 |  | 116.4 | | 45.2 | 27.8 | 205.1 |  |
|  | PPT^a^ (kPa) | | 178.5 | 56.7 | 67.5 | 289.6 |  | 290.3 | | 91.3 | 111.4 | 469.2 |  |
| female | CPT (℃) | | 16.1 | 8.8 | -1.2 | 33.5 |  | 13.6 | | 9.3 | -4.7 | 31.9 |  |
|  | HPT (℃) | | 42.6 | 3.8 | 35.3 | 50.0 |  | 41.1 | | 3.8 | 33.6 | 48.5 |  |
|  | MPT (g) | | 112.7 | 45.7 | 23.2 | 202.2 |  | 98.7 | | 65.4 | NA^d^ | 226.8 |  |
|  | PPT^b^ (kPa) | | 156.6 | 58.4 | 42.1 | 271.1 |  | 256.7 | | 94.8 | 70.9 | 442.5 |  |

*^a^testing site on masseter muscle; ^b^testing site on thenar eminence; ^c^upper limit exceeds baseline temperature, i.e., 32℃; ^d^lower limit exceeds 0g.*

*V1: the first branch of the trigeminal nerve dermatome, T1: the first thoracic nerve dermatome, SD: standard deviation, CI: confident interval, CPT: cold pain threshold, HPT: heat pain threshold, MPT: mechanical punctate pain threshold, PPT: pressure pain threshold, NA: not applicable*
